# The Impact of Intermediate Goods Imports on Energy Efficiency: Empirical Evidence from Chinese Cities

**DOI:** 10.3390/ijerph192013007

**Published:** 2022-10-11

**Authors:** Yu Xiang, Jing Zheng, Xunhua Tu

**Affiliations:** 1 School of Economics, Sichuan University, Chengdu 610065, China; 2 School of Public Finance and Taxation, Southwestern University of Finance and Economics, Chengdu 611130, China

**Keywords:** intermediate goods imports, sustainable development, urban characteristics, energy efficiency, mechanism of action, spatial spillover

## Abstract

Improving energy efficiency is a critical way to solve energy shortage and environmental problems and achieve the goal of “double carbon”. As China expands imports and integrates into global value chains, can import trade improve energy efficiency? This topic is extremely important for solving current energy problems and promoting sustainable economic development. Based on panel data of prefecture-level cities in China, this paper uses the Super-SBM model to measure the total factor energy efficiency of cities and investigates the impact of intermediate goods imports on energy efficiency with fixed effects models and instrumental variable method (IV). The study finds that: (1) intermediate goods imports contribute to the increase of urban energy efficiency, and the mechanism test indicates that intermediate goods imports affect energy efficiency through the technology spillover effect and intermediate goods type diversification effect. (2) According to the heterogeneity analysis, the effect of intermediate goods imports on energy efficiency is more evident in eastern China and cities with low topographic relief, medium population scale, and high absorption capability. (3) Analysis of the spatial spillover effect with the SDM model shows that importing intermediate goods promotes energy efficiency in local cities and radiates energy efficiency improvement in neighboring cities.

## 1. Introduction

As the global economy grows, global warming and climate change have become increasingly critical issues in the past decades [1,2]. According to BP’s 2022 World Energy Statistics Yearbook, global carbon emissions increased by 5.7% year-on-year in 2021; among them, the emissions generated from energy use increased by 5.9% and energy demand increased by 5.8%. The world is still in a carbon growth cycle, thus, it remains challenging to eliminate our dependence on fossil fuels and energy shortages may occur in the future [3], which has become a significant challenge for all countries [4,5].

Since the reform and opening up, the rough development model led to the rapid economic growth of China, making China the second largest economy but also the largest energy consumer [6]. According to the National Bureau of Statistics of China, China’s total primary energy production was 4.33 billion tons of standard coal in 2021, increasing by 6.2% year-on-year. Although China’s total energy reserves are among the highest in the world, its per capita energy reserves are significantly below the global average. With China’s rapid economic development, industrialization and urbanization have accelerated, and the economy has become increasingly energy-dependent, the contradiction between the supply and demand of energy is becoming more and more prominent. On the one hand, China’s economy is undergoing structural adjustment and the economic development model is converting from a quantity-oriented pattern to a quality-oriented one, while environmental regulations are becoming more stringent. On the other hand, coal has both price and reserve advantages, making it impossible for the country to change its coal-based energy structure in the short term. In 2020, President Xi Jinping announced China’s carbon peak target and carbon-neutral vision to the world. Under the constraints of carbon peak and carbon neutral targets, China’s socio-economic development will be under tremendous pressure to reduce emissions. More efficient utilization of resources and reduction of environmental pollution have become important objectives, economic development of the country transit to a resource-saving and environmentally friendly model. Improving energy efficiency becomes the most feasible and realistic means of achieving green economy goals in the short term [7]. Progress in technology contributes to energy efficiency improvement, and foreign trade is an essential source of technological progress and a key factor in promoting technological progress.

China’s international trade has experienced tremendous growth over the past 40 years. The total trade volume has grown from US$20.64 billion in 1978 to US$6.05 trillion in 2021, and its international market share has increased from less than 1% to 13.5%, making it the world’s largest trading country. There has been a growing concern about globalization’s impact on the environment due to the increase in international trade. But for a long time, the role of import trade has mainly been ignored. With China’s accession to the WTO in 2001, significant reductions in import tariffs have contributed to the expansion of imports. Increasing imports plays an essential role in transforming the mode of economic growth. Intermediate goods imports are an essential component of global trade, providing a means for building China into a trade power [8]. 

Between China’s accession to the WTO in 2000 and 2021, UN COMTRADE statistics indicate that imports of intermediate goods accounted for 70 percent of the total goods imports. Intermediate goods are China’s largest import category since its accession to the WTO. Since intermediate goods embed the technology of exporting nations, which makes trade in intermediate goods a vital form of technology diffusion and transfer [9], as a materialized technology spillover [10], it promotes the technological development of importing countries, leading to changes in environmental conditions and energy use. So the environmental effects of intermediate goods imports have been of interest to academic circles. The environmental impact of international trade has been confirmed by several studies [11,12,13,14].

Empirical research on the impact of trade on energy performance at the city level is scarce and mainly focuses on export and the total trade volume. For instance, Gordon [15], Plourde [16], Antweiler et al. [17], and He and Huang [18] argued that trade could increase energy efficiency or decrease energy intensity. Some studies have analyzed the relationship between international trade and energy use from the emissions viewpoint [5,19]. Several scholars have studied the impact of imports on energy consumption. Huang found that imports decrease energy intensity. Mimouni and Temimi [20] argued that import trade could improve energy efficiency through a system of encouragement and elimination in the industrial sector. Previous empirical work shows that trade in intermediate goods improved energy performance at the firm level in Brazil [21], Mexica [22], India [23], and Indonesia [24,25]. 

This paper aims to explore whether and how intermediate goods imports impact energy efficiency at the city level. Adopting the Super-SBM model, we assess the total factor energy efficiency of the cities covering the period 2000 to 2016; based on previous experience, instrumental variables are constructed to identify the causal effect of imports of intermediate goods on urban energy efficiency. Our benchmark findings suggest that imports of intermediate goods increased the energy efficiency during the sample period, and the finding still holds after a series of robustness tests. The mechanism analysis reveals that intermediate goods import promotes energy efficiency through the technology spillover and type diversification effects. 

From the perspective of urban characteristics, the energy effect of imported intermediate goods is more significant in eastern China—areas with low topographic relief, large and mega cities, and areas with high human capital and high R&D investment, but there is no obvious contrast in southern and northern regions and whether they are resource-based cities or not. In addition, due to the extensive spatial correlation of economic activities, we use a spatial Durbin model to investigate the spatial spillover effects of intermediate goods imports on urban energy efficiency, and the results indicate that imports of intermediate goods not only contribute to local energy efficiency improvement but also radiate improvements in neighboring cities.

Our work bridges the gap in the existing literature regarding the theoretical analysis and empirical testing of energy efficiency issues at the city level in China, which carries important implications. Compared to established studies, this study has a three-fold contribution: (1) using city-level data to examine the impact and mechanisms of intermediate goods import on energy efficiency, filling the research gap in the field of intermediate goods import and energy efficiency, and providing new empirical evidence for improving urban energy efficiency under open economy conditions; (2) based on the urban attributes, we explore the differential impact of intermediate goods import on urban energy efficiency in respect of geographic locations, topographic features, resource endowments, population sizes, and absorption capability, providing targeted suggestions for the energy policy of Chinese cities according to local conditions under the new situation; (3) we examine its spatial spillover effects with Spatial Durbin Model, providing a decision-making basis for building regional joint prevention and control mechanisms.

The remainder of this paper is organized as follows. Section 2 is a literature review. Section 3 presents the empirical strategy, variables, and data information.Section 4 summarizes and discusses empirical results, and Section 5 provides the conclusions

## 2. Literature Review

### 2.1. Measurement of Energy Efficiency

In 1995, The World Energy Council defined energy efficiency as “the reduction of energy inputs to ensure equal energy output”. Patterson (1996) [26] proposed a definition of energy efficiency as “the use of less energy to produce the same number of services or useful output”. Subsequently, D. Bosseboeuf et al. (1997) [27] expanded the traditional definition of energy efficiency and argued that energy efficiency should be understood in terms of both economic and technological aspects—the economic aspect referring to obtaining more output while consuming less energy and technological aspect referring to reducing energy consumption reduction through technological advances and changes in lifestyle and other factors. Furthermore, environmental factors are similarly critical reference indicators for energy efficiency assessment, which are primarily used to evaluate pollutants released from energy consumption. 

The total factor energy efficiency is the mainstream method for energy efficiency evaluation at present, including two types of parametric and nonparametric methods. Stochastic frontier analysis (SFA) represents a parametric method and is a primary method for dealing with multiple-input single-output models; many scholars have applied it to evaluate energy efficiency in various countries [28,29,30,31]. SFA methods cannot handle a model with multiple inputs and multiple outputs. Nonparametric methods such as data envelopment analysis (DEA) have been widely used in energy efficiency assessments to solve this problem.

Hu and Huang (2006) [32] were the first to use DEA method to measure the total factor energy efficiency of Chinese provinces during the period between 1995 and 2002, and defined energy efficiency as “the capacity to maximize output for a given energy input or minimize energy input for a given output”. Tone and Tsutsui (2009) [33] proposed a non-radial, Slack-based SBM(slacks-based measure)and DSBM(dynamic slacks-based measure) model based on the CCR(variable returns to scale,) and BCC(constant returns to scale) models, effectively solving the problem of radial constraints on the input factors, thus reducing the measurement error and making the results more accurate.

### 2.2. The Relationship between International Trade and Energy Efficiency

In recent decades, the relationship between international trade and the environment has been extensively discussed due to the rapid development of international trade [34]. Several studies have explored the environmental impacts of trade, but the majority have concentrated on the effects of international trade on pollution emissions [13,14,35]. In contrast, there have been few studies on the impact of international trade on energy efficiency.

Some scholars have studied the relationship between international trade and energy efficiency using cross-country data, but no consistent conclusions have been drawn. According to Gordon (1993) [15], the increased market size following trade liberalization enabled energy efficiency to increase due to increased trade between the United States, Canada, and Mexico. Using natural gas trade data from 1967 to 1992 in Canada, Mexico, and the United States, Plourde (1993) [16] found that trade liberalization affects the industrial structure, reduces market distortions, and increases energy use efficiency. Antweiler et al. (2001) [17] attributed the environmental effects of trade to technology effects, Scale effects, and composition effects. He and Huang(2021) [2] suggested that export trade could lead to the energy efficiency improvement of firms, and trade liberalization facilitated the improvement of firms’ environmental performance; they put forward a new mechanism of action—innovation investment. Using G7-countries data from 1996–2015, Shah et al. (2022) [36] found that trade is an effective channel to improve energy, and there is a bilateral relationship between energy efficiency and international trade. He and Huang (2020) [18] argued that processing exports could promote firms’ energy efficiency through innovation effect and cost reduction effect in the Chinese manufacturing industry. Zhao and Lin (2020) [37] empirically analyzed whether foreign trade affects energy efficiency in the textile industry of china demonstrated positive feedback between foreign trade and energy efficiency in the textile industry, with imports impacting energy efficiency more than exports. Based on the theory, Cole (2006) [38] found that a rise in international trade scale stimulates aggregate demand and increases domestic production, which increases energy consumption, i.e., international trade increases energy intensity through Scale effects.

Regarding the relationship between import trade and energy efficiency, few scholars have discussed the cause and effect directly. Several scholars have studied the impact of imports on energy consumption; more specifically, by using panel data of China’s 30 provinces from 2000 to 2013, Huang et al. (2017) [39] found that technology spillover coming from the openness of foreign direct investment and import decreased energy intensity. Using firm-level data on manufacturing firms in Indonesia covering the time period 1991 to 2005, Imbruno and Ketterer (2018) [25] conducted theoretical and empirical research on whether the import behavior of intermediate goods impacts firms’ energy consumption, and found that firms that import intermediate goods consume 13.9% less energy than non-importing firms. Constructing firm-specific and time-varying measures of output tariffs faced by Mexican firms during the 2000–2003 period, Gutiérrez and Teshima (2018) [22] explored whether tariff changes affect energy efficiency, abatement expenditures and pollution, and found that import competition lead to an increase in plants’ energy efficiency, but a decrease in abatement expenditures, the main driver of the increases in energy efficiency is very likely a change in cost-cutting practices and improvements in technical efficiency. Using a sample of 100 countries from 1980 to 2015, Mimouni and Temimi (2018) [20] investigated the impact of Foreign Direct Investment (FDI), imports, and industry value-added on energy efficiency and argued that the competitive market effects of import trade could improve energy efficiency through a system of encouragement and elimination in the industrial sector.

However, some studies put forward completely different opinions. Chen et al.(2022) [40] estimated the energy intensity of 30 provinces in China from 2005 to 2018, and explored the impact of trade openness and economic growth on China’s energy intensity, they found that the effects of foreign trade on energy intensity is primarily attributed to the export route, while the import route has a negligible effect. Using macro data for eight Middle Eastern countries from 1980–2007, Sadorsky (2013) [41] found that import trade negatively correlated with energy use efficiency. Yao et al. (2021) [42] examined the energy efficiency of 36 countries around the world and found that the value added of exports had a much greater impact on energy efficiency than the value added of imports.

To sum up, existing studies have documented the impact of international trade on energy efficiency, but most of these studies are based on industry and firm perspectives, as well as national, regional, and provincial levels. However, few studies have been conducted on energy efficiency and its influencing factors at the city level, moreover, several studies are basically from the perspective of total trade and export trade, and the impact of import trade on this is often neglected. Therefore, in the context of China’s import expansion strategy, it is crucial to examine the impact of intermediate goods imports on urban energy efficiency, complementing the research on import trade and environmental energy issues.

## 3. Data and Model

### 3.1. Data Sources and Processing

The trade data in this paper is derived from the China Import Customs Database, which covers the period 2000–2016. The data includes information such as enterprise code, import and export type, transaction amount, product HS (The Harmonization System) Code, city, and destination, and trade type. 

The data were processed according to the research needs of this paper: First, only keep the data of general trade, processing imported materials, processing materials supplied by clients, and assembly trade. Second, considering the inconsistency of HS codes used in the customs data before and after 2002, HS codes were adjusted according to the HS 6-digit code conversion codes provided on the United Nations website, and all data were integrated into the HS 6-digit code level. Third, identified imported intermediate goods by using United Nations HS codes in conjunction with BEC (Broad Economic Catalogue) mapping tables [43]. Finally, the enterprise data was aggregated to the city level to obtain the urban data of the intermediate goods trade. The study excluded cities in Tibet Autonomous Region, Taiwan Province, Hong Kong Special Administrative Region, and Macao Special Administrative Region due to data availability; in addition, cities with severe missing data, such as Tongren and Liupanshui, were excluded, leaving a panel data of 272 prefecture cities in China. Urban data are obtained from the China Statistical Yearbook, China City Statistical Yearbook, China Environmental Statistical Yearbook, National Research Network, and China Economic Network database, etc. A few missing data were supplemented and improved through interpolation.

### 3.2. Econometric Model 

Based on the existing literature, we adopt a two-way fixed effect model to empirically study the impact of the intermediate goods imports on energy efficiency:

(1) $$EE_{it}=\alpha_{0}+\alpha_{1}lninter_{it}++\beta Control_{it}+u_{i}+v_{t}+\varepsilon_{it}$$

where *i*, *t* represent the city and year, respectively, and $EE_{it}$ denotes the energy efficiency of city, *i* the current year—the exact measurement process will be described below. The core explanatory variable $lninter_{it}$ is the logarithm of imports of intermediate goods; the $Control_{it}$ are the control variables, $u_{i}$, $v_{t}$ and $\varepsilon_{it}$ represents the city fixed effects, year fixed effects, and random disturbance terms, respectively.

The expansion of imports in one city is likely to affect the economic activities of other cities through upstream and downstream links in the industrial chain. Therefore, ignoring spatial spillover effects results in estimation bias. In this paper, we introduce a spatial econometric model to test the spatial spillover effect of intermediate goods imports; referring to Elhorst (2010) [44], we use the Spatial Dubin Model (SDM) as the initial model.

(2) $${\mathrm{EE}}_{i,t}=\alpha_{0}+{\rho \mathrm{WEE}}_{i,t}+\alpha_{1}{\mathrm{lninter}}_{i,t}+{\beta \mathrm{Wlniter}}_{i,t}+\alpha_{k}\;Control_{it}\;\;\;\;\;\;\;\;+\delta WControl_{it}+u_{i}+v_{t}+\varepsilon_{\mathrm{it}}$$

where, W is the spatial weight matrix and $\;{\rho \mathrm{WEE}}_{i,t}$ is the spatial lagged term of the explanatory variable, indicating the effect of energy efficiency in neighboring regions on the region, ρ is the spatial autocorrelation coefficient, β is the spillover effect from intermediate goods imports, and δ is the spatial spillover effect of the control variables.

Three spatial weight matrices are constructed in this paper based on the characteristics of the city’s geography and economic activity: (1) the geographical distance matrix $W_{1}$, using the inverse of the distance between geographies as weights, i.e., $W_{i,t}=\frac{1}{d_{ij}}$, $d_{ij}$ is the geographical distance between the two cities, which is calculated based on each city’s latitude and longitude information. (2) Spatial matrix of economic distance $W_{2},$ constructed at the inverse of the absolute value of the difference in real GDP per capita. (3) Economic geography nested matrix $W_{3}$, incorporating both geographic distance and economic distance, calculated as: $W_{3}=1(-\varphi )W_{1}+W_{2}$, φ varies from around 0 and 1, and this paper takes the value 0.5.

### 3.3. Variables

#### 3.3.1. Explained Variables

Total factor energy efficiency, hereafter referred to as energy efficiency (EE), was measured using the undesirable super-efficiency SBM model; input–output indicators are described in Table 1. Labor, capital, and energy as inputs, urban gross domestic product as desired outputs, and emission of sulfur dioxide and carbon dioxide as non-desired outputs. 

In terms of data processing: (1) Real GDP was calculated by using the year 2000 as the base year. Urban capital stock was calculated using the perpetual inventory method,$\;K_{t}=K_{t-1}\left(1-\delta \right)+I_{t}$ and the depreciation rate *δ* is set to be 9.6% [45], $I_{t}$ represents the current social fixed asset investment after deflation; the capital stock for the based year (2000): $K_{2000}=I_{2000}\left(\delta +g\right)$, *g* is the geometric average growth rate of GDP of each city during the research period, $I_{2000}$ is the fixed asset investments in 2000. (2) Referring to Li et al., (2016) [46], urban energy consumption includes total annual electricity consumption and liquefied petroleum gas consumption, the data are obtained from the Chinese City Statistical Yearbook published by the National Bureau of Statistics for all years. (3) CO_2_ emissions were estimated using the particle swarm optimization-back propagation (PSO-BP) algorithm and summed up to city-level [47], SO_2_ emissions are obtained from the Chinese City Statistical Yearbook.

**Figure 1:**
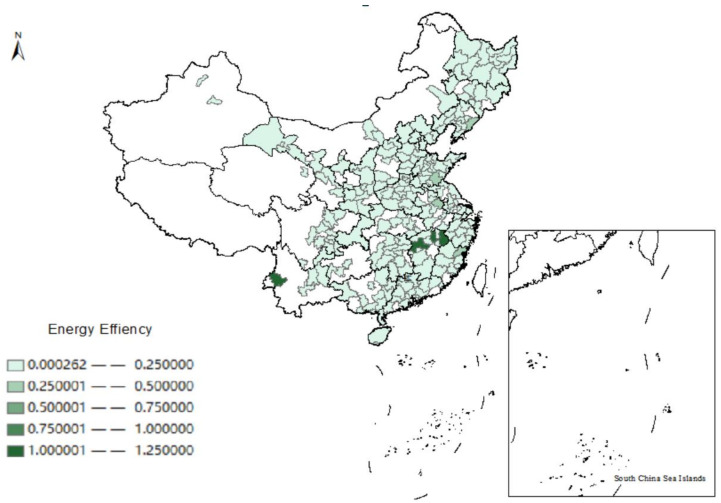
Spatial distribution of energy efficiency in China in 2000.

**Figure 2:**
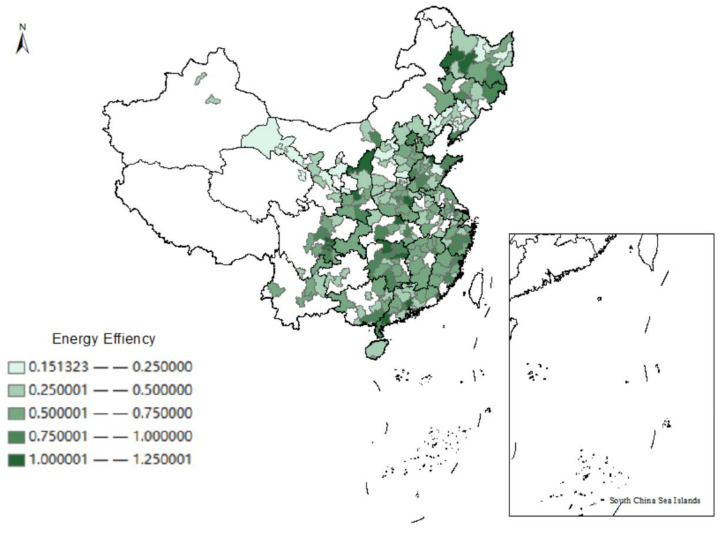
Spatial distribution of energy efficiency in China in 2016.

Based on the measurement results, we use ArcGIS software to depict the spatial distribution of the energy efficiency of cities of China in 2000 and 2016, as shown in Figure 1 and Figure 2. The energy efficiency of Chinese cities has improved significantly over a dozen years; the majority of the Chinese cities were at a low level of energy efficiency (EE < 0.25) in 2000. However, the number of cities at low energy efficiency is diminishing over time, with most of them hitting medium and even above levels (EE > 0.50) in 2016. Meanwhile, we notice that the energy efficiency in China’s eastern coastal regions is particularly high. The rapid economic development and high-level industrial agglomeration in these areas may explain this phenomenon [48].

#### 3.3.2. Explanatory Variables

Urban imports of intermediate goods ($lninter_{it}$), we take the logarithm of imports of intermediate goods summed at the city level.

#### 3.3.3. Control Variables

Economic development (lnpgdp) is expressed as the logarithm of GDP per capita, foreign investment (lnfdi) is expressed as the logarithm of the actual amount of foreign direct investment utilized by each city, urbanization (urban) is expressed as the logarithm of population density, financial development (fina) is expressed as the ratio of the loan balance of financial institutions to GDP, and infrastructure (RF) is expressed as the amount of road freight.

## 4. Results and Discussion

### 4.1. Benchmark Regression

In this section, we aim to explore whether intermediate goods imports have an impact on the energy efficiency of cities. By importing new machinery equipment to replace old ones (where new technologies are typically less energy intensive than what is already available in the local area), intermediate goods imports help reduce energy consumption. In addition, a great deal of previous literature has demonstrated that intermediate goods imports significantly promote technological innovation and R&D [11,49,50,51]. Aside from that, the induced competition caused by imports forces locally inefficient firms to become more productive, thereby improving their energy efficiency [23,51,52,53]. Therefore, we propose a hypothesis that imports of intermediate goods contribute to energy efficiency improvement.

The energy efficiency calculated in Section 3.3 is taken as the dependent variable, and the fixed effects model is applied to analyze it. Table 2 presents the results of the benchmark regressions. Column (1) contains neither control variables nor control for year and city fixed effects; in column (2), control variables are added; in column (3), year fixed effects are added; and in column (4), city fixed effects are added. As can be seen, the effect of intermediate goods imports on energy efficiency is significantly positive in each model, indicating that imports of intermediate goods can effectively improve urban energy efficiency, which is consistent with our theoretical hypothesis. Considering the possible lag effect of the economic activity, the regression results of the explanatory variable lagged by one period (L.lninter) are presented in column (5), and the results show that the positive effect of intermediate goods imports is still significant.

### 4.2. Quantile Regression

The previous benchmark regression mainly depicted the impact of intermediate goods imports on urban energy efficiency at the average level. In order to further analyze the effects of intermediate goods imports on different energy-efficient cities, this paper uses a panel quantile regression model and selects five quantile points of 0.1, 0.25, 0.5, 0.75, and 0.9 for estimation. As shown in Table 3, the estimated coefficients at different quantile points all pass the significance tests, indicating that intermediate imports’ impact on energy efficiency is universal. Estimates of regression coefficient increase as the quantile point increases, indicating that the upgrading effect of imports of intermediate goods is more significant in high-efficiency regions than in low-efficiency regions.

### 4.3. Robustness Tests

#### 4.3.1. Endogeneity Issues

Intermediate imports may be linked to energy efficiency by two-way causality, in addition, missing variables and measurement deviations may cause endogeneity problems. The instrumental variables approach is used in this study in order to mitigate as much as possible the endogeneity problem caused by reverse causality and omitted variables, etc. Referring to Yu (2015) [54], we use the weighted intermediate import tariff rates of intermediate goods at the city level as an instrumental variable to address the endogeneity issue, referring to Erten et al.(2019) [55], the index of import tariff of intermediate goods at the city level is constructed as follows:

(3) $$TC_{it}=\underset{j}{\sum }\frac{I_{jit}}{I_{ji}}Traiff_{jt}$$

where *i* denotes city, *t* denotes year, and *j* denotes HS product. $Traiff_{jt}$ denotes the tariff rate of HS product *j* in year *t*,$\;I_{jit}$ denotes the value of imports of product *j* in city *i* in year *t*. $I_{ji}$ denotes the value of intermediate import in city *i* in year *t*. As seen in Equation (2), the city-level intermediate import tariff is a weighted average of the product-level tariff, and the weight is the share of the value of the HS product in the total intermediate goods imports in the city. Tariff data are obtained from the WITS.

Moreover, considering that China’s imports exhibit a spatially decreasing pattern from the eastern coast to the interior regions, we use the distance from cities to the nearest port as an instrumental variable for intermediate goods imports. More than 80% of international trade in goods is conducted by sea currently, the cost of transportation between cities and ports makes a significant portion of their trade costs, which is highly related to the trade volume. Since it is cross-sectional data, the interaction term of the distance and the exchange rate between the USD and the RMB yearly is another instrumental variable; the exchange rate are obtained from the World Bank database.

Table 4 reports the regression of the two-stage least square method(2SLS). In particular, Column (1)–(2) list the estimated results of import tariff data on intermediate goods as instrumental variables. Columns (3)–(4) present the results of using the distance from the nearest port as an instrumental variable. Columns (1) and (3) show the results of the first stage regression, and the results show that the instrumental variables pass the 1% statistical significance level test, demonstrating the relevance requirement of the instrumental variables, tests of Kleibergen-Paap rk LM statistic and Kleibergen-Paap Wald rk F-statistic reject the original hypothesis that the instrumental variables are underidentified or weak. In columns (2) and (4), imports of intermediate goods are significant at 1%. Thus, after controlling endogeneity issues, the estimation results are relatively consistent with the results from previous benchmark regressions; hence the results are mainly reliable.

#### 4.3.2. Other Robustness Tests

We use the following methods for robustness testing:

(1) Replace the explanatory variable indicator. We use the Partial Factor Energy Efficiency (PFEE) substitute for EE; PFEE is a city’s ratio of effective output and energy input. The results in column (1) of Table 5 show that imports of intermediate goods significantly boost Partial Factor Energy Efficiency at the 1% significance level, supporting the findings of the benchmark regression.

(2) Excluding macro-systematic differences, the robust standard errors of the benchmark regressions are clustered only at the city level. We exclude the effects of macro-systematic differences by controlling for “province-year” joint fixed effects and clustering the standard errors at the province and year levels, column (2) shows that the energy efficiency effect of intermediate goods imports remains robust at the 1% level.

(3) Data tailing, to minimize the impact of outliers on the empirical results, from the perspective of data robustness, the regression is re-run with the tail of all indicators reduced by 1%. Column (3) shows that the significance level of the estimates has not changed after excluding other policy effects.

(4) Excluding the impact of energy policies. In recent years, the Chinese government has continued to promote “energy saving” and “emission reduction”; following the issuance of the “Notice on the Pilot Program of Energy Conservation and New Energy Vehicles” on January 2009, a total of 26 cities have been selected in three batches. Since June 2011, 30 cities have been selected by the National Development and Reform Commission in three batches to participate in the comprehensive demonstration cities for energy conservation and emission reduction fiscal policies. We exclude these cities from the sample to minimize the effects of relevant energy policies. The estimates are presented in column (4) and suggests that the significance level of the estimates has not changed after excluding other policy effects.

### 4.4. Mechanism 

In this section, we explore the mechanisms through which intermediate goods imports improve urban energy efficiency. On the one hand, the imports of intermediate goods produce technology spillovers that can increase technological innovation within a country [56], and technological advances are crucial to energy efficiency [57]. On the other hand, intermediate goods imports enrich the types of intermediate inputs, facilitating market competition and enabling companies to focus more on technological R&D and quality management, ultimately resulting in an increase in energy efficiency [2]. Therefore, we propose another hypothesis that intermediate goods imports promote urban energy efficiency through technology spillover and type diversification effects.

To test whether intermediate goods imports can affect energy efficiency through “technology spillover effect” and “types diversification effect”, we use the interaction terms lninter × lnspillover, lninter × types to empirically test how intermediate goods imports affect urban energy efficiency through the above mechanisms.

Technology spillovers from the import of intermediate goods are measured through the L-P model proposed by Lichtenberg and Pottelsberghe (1998) [58]; the first step is to calculate the stock of foreign R&D capital acquired by China through import channels in year *t*, which is known as imported technology spillover:

(4) $$S_{it}^{f}=\sum \frac{M_{ijt}}{Y_{jt}}S_{jt}^{d}$$

$Y_{jt}$ is the GDP of country *j* for the current year, $M_{ijt}$ is the amount of intermediate goods imported by China from country *j* of city *i*, $S_{jt}^{d}$ denotes the domestic R&D capital stock of country *j* in year *t*, calculated by the perpetual inventory method, according to Griliches (1998) [59]:

(5) $$\;S_{jt}^{d}=\left(1-\delta \right)S_{jt-1}^{d}+RD_{jt}$$

(6) $$S_{j2000}^{d}=\frac{RD_{j2000}^{d}}{\delta +g_{j}}$$

$S_{jt-1}^{d}$ denotes the domestic R&D capital stock of country *j* in the previous year, the year 2000 is the based year, $S_{j2000}^{d}$ is the R&D capital stock in country *j* in 2000, $RD_{jt}$ is the R&D capital investment, $g_{j}$ is the average annual growth rate of R&D investment, δ is the depreciation of the R&D capital stock, which is set to be 5%, according to Coe and Helpman (1995) [60]. The data of R&D and GDP for countries are obtained from the World Bank and UN TRADE databases.

For the metric for the types of intermediate goods, Strauss-Kahn (2011) [61] treats identical products from different countries as different products. That is, the same product should not only be the same HS code, but the source of import should also be the same. We employ the logarithmic form of the variable. 

As shown in Table 6, column (1) shows that the interaction term lninter × spillover is significantly positive, indicating that the intermediate goods imports lead to international technology spillover and contributes to urban energy efficiency: the reasons are as follows: First, intermediate goods imports with advanced technology and knowledge is equivalent to technology transfer [23], which importers can imitate and learn from, and then invest more in R&D to improve their technology [56]. Second, technical guidance, after-sales services, or expert assignment provided by the exporters can indirectly improve the technical knowledge reserve of the imported enterprises, accelerate the digestion and absorption of the technology, improve their production processes and equipment utilization, and thus achieve energy efficiency improvement [62]. Third, increasing intermediate goods will certainly intensify the competitive effect, which may result in the enterprises stepping up technological research and development investments, optimizing resource allocation, and thus improving their technological innovation capabilities, promoting technological progress in the industrial sectors [63]. By improving technology, pollution emissions in production can be effectively reduced [64,65], which is one of the most important drivers of long-term improvements in energy efficiency [60,66]

Likewise, column (2) shows that intermediate goods imports enhance energy efficiency through the types diversification effect; previous studies have found similar results [25]. First, it has been demonstrated that diversifying intermediate goods has a complementary effect to domestic intermediate inputs to improve the efficiency and output of firms by optimizing the allocation of production resources [24]. Second, as the variety of intermediate goods increases, the fierce competition in the domestic intermediate goods market leads to a decline in the price of intermediate goods, leading enterprises to seek more energy-efficient, environmentally friendly, high-quality, and low-cost inputs for intermediate goods, thereby expanding production scale and increasing output. In addition, the diversification of intermediate goods may lead to diversified technologies spilling over into the final product sector, enabling it to choose products that meet greener requirements, reduce resource waste, improve marginal efficiency of production, and optimize enterprise organizational structures and production systems to achieve energy savings and improve energy efficiency. In column (3), we bring both technology spillover and types diversity in the regression, and the result shows that the channel of technology spillover effect is more significant than that of types diversity effect.

### 4.5. Heterogeneity Analysis—The Impact of Urban Characteristics

Significant city disparities exist between China’s eastern, central, and western regions. Even in the same region, each city has its characteristics in terms of natural resources and environment, as well as human factors, such as national policies and local governance [32]. Therefore, to fully realize the role of intermediate goods imports in promoting energy efficiency, China should implement differentiated environmental and energy policies.

#### 4.5.1. Impact of the Geographic Location of the City

- East-Central-West Heterogeneity.

Based on geographical location and level of economic and technological development, primarily due to longitude differences, China State Council has divided China into three major economic zones, East, Central, and West. Most of China’s ports are located along the eastern coast. When maritime transportation is the predominant mode of international trade, longitude may indicate the distance of a region from the coastline and, therefore, the region’s openness to the outside world. Hence, eastern coastal regions are usually more economically dynamic, with flexible social structures that can respond quickly to changes in the external environment.

As shown in Table 7, the results in columns (1)–(3) indicate that intermediate goods imports have a significant positive impact on energy efficiency in the eastern region but are not significant in central and west China. Because of its proximity to the coast and well-developed waterways, Eastern China has the inherent advantages of conducting international and domestic trade, a sound economic foundation, a vibrant market, a highly developed education system [67], and can benefit fully from the improvement in energy efficiency brought about by import of intermediate goods. That is due to the fact that cities in the eastern coastal areas have developed economies with a relatively high level of openness. Meanwhile, they have high levels of industrial integration, advanced science and technology, and ample capacity for innovation [68]; they can give full play to the energy promotion effect of intermediate product import.

In contrast, the central and western cities are located inland, thus resulting in natural conditions and social development inferior to eastern cities, many of which are still at the mid-industrialization stage; economic development is still extensive due to the deficiencies in infrastructure, technology innovation environment, capital supply, talent support, etc. The energy efficiency improvement effect of intermediate goods imports has not yet been realized sufficiently.

- North–South heterogeneity.

Climate conditions such as temperature, humidity, and precipitation differ significantly between southern and northern China, leading to considerable differences in the geographical landscape, production methods, and cultural practices between the two regions, causing differences in industrial development and energy consumption, and the border between South and North is generally based on the boundary proposed by Zhang Xiangwen in 1908. 

The results in columns (4)–(5) indicate that intermediate goods imports have a positive effect on energy efficiency, both in the North and South. But, relative to the south, the improvement is more significant in the North. Perhaps this is due to the fact that resource-intensive heavy chemical enterprises dominate the northern region. Compared to the light industry, heavy chemical enterprises consume more energy and emit more pollutants; the marginal impact of intermediate goods imports on the energy efficiency of heavy chemical enterprises is greater than that of the light industry. 

### 4.5.2. Impact of Urban Topographic Features

The topography of a region not only influence human physiology and behavior, affecting worker productivity, but also influence the choice and scale of industries, affecting economic growth and energy consumption within the region [69]. Relief amplitude is the difference between the altitude of the highest point and the lowest point in a city, and it is a macroscopic index to describe the topographic features of a city; we use it as a proxy variable for urban geographic features [70]. In this study, the Relief amplitude data are trisected into three groups of low, middle, and high.

Under different topographic undulations, the effect of intermediate import on energy efficiency is heterogeneous, as shown in columns (1)– (3) of Table 8. In general, the larger the relief amplitude, the less significant the effect of intermediate imports on improving energy efficiency in the city, and the negative effect of intermediate imports can be attributed primarily to transportation and social production in the city. Compared to mountainous and highland areas, plains and basins have a significant economic cost advantage. Other things being equal, transportation is significantly more difficult on mountainous plateaus than on plains, resulting in greater energy consumption and increased pollution emissions [71].

### 4.5.3. Impact of Urban Resource Endowment

The natural resources that underlie urban development influence energy utilization and pollution emissions in cities. Hence, this study examines the heterogeneous impacts of imported intermediate goods on urban energy efficiency from the perspective of resource endowment. Under the China State Council, resource-based cities are those developed primarily by using natural resources in the local area (e.g., minerals, energy, etc.); these 272 cities can be divided into 108 resource cities and 164 non-resource cities based on this criterion.

Columns (4)–(5) of Table 8 show the regression of resource endowment heterogeneity; it is clear that the urban energy efficiency improvement by intermediate goods imports is very significant in both resource cities and other cities, which reflects the universality of the impact of intermediate goods imports on energy efficiency. Resource-based cities generally have high energy consumption, high carbon emission, and high pollution [72]. Usually, resource-based cities basically rise or grow as a result of the exploitation of natural resources, and resource-dependent industries account for a greater share of their industrial structure; resource-based cities are characterized by high dependence on resources, loose urban spatial structures, and simplicity of industrial structures compared to other cities [73]. The technology spillover from the import of intermediate goods can improve the energy efficiency of resource-based cities, and this realization path is important for promoting the low-carbon transition development of resource-based cities and achieving carbon peaking and carbon neutrality [74]. This demonstrates the important role of intermediate goods imports in the green transformation of resource-based cities.

### 4.5.4. Impact of Urban Population Scale

Rapid urbanization is taking place in China at present, which will have a great impact on the environment and the use of energy [31]. Compared to population agglomeration, changes in total population significantly impact the urban energy environment and carbon emissions [75]. There is a direct link between population and energy production and consumption. In addition, the city’s population is an important factor that reflects the size of the city directly [72]. To verify the heterogeneous effects of urban population on energy efficiency, we group these cities based on the number of permanent residents of each prefecture-level city, according to city scale classification criteria released by the State Council’s 2014.

As shown in Table 9, the impact of intermediate goods imports on energy efficiency varies significantly by population size. Precisely, the impact of intermediate goods imports on urban energy efficiency shows a characteristic shape of a “inverted U”, i.e., the impact of intermediate goods imports on energy efficiency increases as the population rises but decreases after a certain scale. The reason for this is that the technology spillover effect and diversification effect of intermediate goods imports are built on the initial endowment of technical skills, capital, and talent on a specific scale, small and medium cities do not have sufficient infrastructures such as human capital, innovation environment to take advantage of the efficiency improvements brought about by intermediate goods. In large and megacities, the higher factor productivity promotes the efficient use of energy [76]. While super megacities are prone to "urban diseases, "such as traffic congestion and environmental pollution [77], which limit the release of import effects. 

### 4.5.5. Impact of Urban Absorptive Capacity

The intermediate goods imports generate technology spillovers for a country, but the characteristics of the host country may have a major impact on its effects. Cohen and Levinthal [78] defined absorptive capacity as the ability to learn, absorb, and use helpful technology to enhance its output. Subsequently, scholars gradually extended the concept, arguing that absorptive capacity is a product of R&D activities, and human capital is the primary absorptive capacity. Only through high levels of human capital and R&D investment can advanced technologies embedded in imported intermediate goods be effectively absorbed [79]. 

We use the number of college students per 10,000 individuals to indicate the human capital and the ratio of science and technology expenditure to GDP to indicate the investment in research and development (R&D). the two indicators are trisected into three groups of low, middle and high. Regressions in groups are shown in Table 10; columns (1)–(3) reveal a significant difference in the energy efficiency enhancement effect of intermediate import at different levels of human capital; the energy efficiency enhancement effect is more significant with the increase of human capital. It indicates that the agglomeration of high-quality human capital is more helpful in promoting the improvement of regional energy efficiency. With increased levels of human capital, energy efficiency enhancement is more significant.

Columns (4)–(6) show the effect of different R&D inputs on the effect of energy efficiency by intermediate goods imports. When only at the high level of R&D, the energy efficiency effect is significant. The higher R&D investment, the higher the ability to update energy efficiency and emission reduction technologies, and the more it helps to improve urban energy efficiency. The more R&D investments uncover the fact that it reflects the importance enterprises attach to the development of technological innovations and their level of technological development [37]. They can develop more advanced production technologies and more energy-efficient management methods by increasing the funds and materials devoted to technology research and development, encouraging technological advancement, and improving energy efficiency, which is consistent with Huang et al. [39]. The impact of technology spillovers from imported goods on energy efficiency is affected by the level of local R&D expenditure and personnel, which enables us to develop a variety of policies and measures to promote more energy efficiency.

## 4.6. Spatial Spillover Effects of Intermediate Goods Imports on Urban Energy Efficiency

The First Law of Geography states that everything is interconnected, and the closer the distance, the stronger the connection. Regional economic activities are also spatially correlated, with one city’s economic and trade activities linked to those in surrounding areas, which in turn may affect energy use and pollution emissions in surrounding areas. Intermediate goods imports have spillover effects on economic activities in neighboring regions. Firms in the surrounding areas can access advanced technology from imported intermediate products at a lower cost. The development of cross-regional markets contributes to the rational allocation of resources and technologies within and between regions. It enhances regional technology development and production efficiency, thereby improving regional energy efficiency [80]. Therefore, we propose the hypothesis that there is a strong spatial spillover effect of intermediate goods import, which not only enhances the local energy efficiency but also creates a strong radiation-driven effect on the surrounding regions.

### 4.6.1. Test of Spatial Correlation

**Figure 3:**
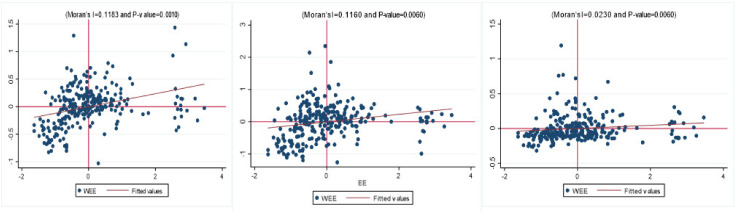
Moran scatter plot of EE for three matrixes (W_1_, W_2_, W_3_).

Spatial autocorrelation is a prerequisite for using spatial econometric models. We use the global Moran’s I to test the spatial autocorrelation for urban energy efficiency (EE). Table 11 shows that, with the exception of the initial years, the global Moran’s indices of energy efficiency (EE) pass the 1% significance level test, indicating that spatial spillover effects of energy efficiency of cities in China are significant and positively spatial correlated. To further analyze spatial correlation patterns across cities, this paper uses Stata to calculate the local Moran index and draws Moran’s scatter plot for 2016 to reveal the local autocorrelation of energy efficiency. As seen in Figure 3, most cities in China are within quadrant 1 and quadrant 3, indicating a positive autocorrelation in energy efficiency for each city.

### 4.6.2. Spatial Econometric Model Estimates

According to Elhorst (2014) [81], we use Wald and LR tests to select appropriate spatial models from specific to general and then from general to specific. As shown in Table 12, all spatial errors and spatial lag multipliers significantly reject the original hypothesis under three different spatial matrices, so the spatial durbin model (SDM) is superior to spatial lag model (SAR)and spatial error model (SEM). We also report the estimation of the spatial lag model (SAR) to compare the robustness of the estimation results. We next measure both direct and indirect marginal effects on energy efficiency.

As shown in Table 12: no matter which spatial weight matrix is used, the overall effect of the econometric model on the energy efficiency of intermediate goods imports is significantly higher than that estimated by the benchmark regression model. There is a significant positive direct effect of intermediate goods imports not only on energy efficiency in the city, but also on neighboring cities, and the indirect effects are all larger than the direct effects. The overall effect is significant, indicating that the import of intermediate products has a positive impact on the energy efficiency of the city and is conducive to the formation of a regional green co-development patter. According to these results, integrated energy planning should consider both local impacts as well as spillover effects generated by adjacent regions in a systematic and holistic manner.

## 5. Policy Implications

To effectively improve urban energy efficiency, we bring relevant countermeasures and suggestions based on the empirical results.

Firstly, continuously implement an active import promotion strategy to fully exploit the function of intermediate goods imports to promote urban energy efficiency. Generally speaking, imports of intermediate goods can significantly enhance the efficiency of urban energy use. Due to this, the Chinese government should recognize the importance of importing intermediate goods to enhance energy efficiency, formulate effective policies, and adopt positive measures to encourage import; in this way, bridge the technology and efficiency gaps, capture technology spillovers, diversify the types of intermediate goods inputs and improve energy efficiency ultimately.

Secondly, advance urbanization rationally, value regional differences, and formulate relevant policies according to local conditions. In order to ensure that the intensive function of urban development is fully realized, it is necessary to regulate its expansion reasonably to avoid environmental and energy problems caused by overpopulation. The impact of natural environmental factors on regional energy efficiency is significant, a reasonable balance should be struck between the development status of each region in formulating targeted policies on intermediate goods imports due to the disadvantages of the economic development base and import trade development in the western region, as well as the complexity of the geographical environment. It is necessary to strengthen the policy support for opening up the regions to the outside world, especially by relying on the "Belt and Road" initiative and the "opening up to the west" strategy to activate the development of the central and western regions. 

Thirdly, strengthen human capital accumulation, increase R&D investment, and continuously cultivate the absorptive capacity. The international technology spillover transmitted by intermediate goods imports can only contribute to the improvement of energy efficiency if it is fully digested, absorbed, and transformed. Therefore, it is necessary to enhance the absorption and utilization of technology spillover, and China should vigorously optimize the structure of R&D investment, improve the digestion and transformation capacity, and adapt and localize the external technology digested and absorbed. Moreover, the local government should pay attention to cultivating talents and introducing high-quality talents to achieve human capital accumulation.

Fourthly, we should give full play to the spatial spillover effect of intermediate goods imports on energy efficiency, establish joint control mechanisms across regions, including coordination of pollution control actions and environmental policy design across the areas, and avoid beggar-thy-neighbor governance disorder. In addition, barriers limiting factor flows, such as local protection and market segmentation, should be weakened. Regions need to strengthen infrastructure sharing and the flow of talent and information and expand the borders of spillover regions.

## 6. Conclusions

Import expansion is crucial in transforming the economic growth mode and further promoting high-quality development. The relationship between energy efficiency and intermediate goods imports is significant for the environment and economic development. In the context of China’s active importing strategy, based on the fixed effect model and moderating Effect models, this paper systematically investigates the impact of China’s intermediate goods imports on urban energy efficiency and its mechanism by adopting the prefectural-city panel data of China from 2000 to 2016. In addition, the urban heterogeneity and spatial spillover effect are further discussed.

It is found that: (1) intermediate goods imports have a significant positive impact on urban energy efficiency; the regression obtained by using the instrumental variables approach and other robustness tests still confirm this conclusion. Mechanism tests verify that intermediate goods imports promote urban energy efficiency through technology spillover and type diversification effects. (3) Heterogeneity analysis show that the energy effect of intermediate goods imports is more significant in eastern China, low relief amplitude areas, large and megacities, and cities with high human capital and high R&D investment. However, there is no difference between the southern and northern regions and whether they are resource cities. (4) Analysis of the spatial spillover effect shows that importing intermediate goods not only promotes the energy efficiency of local cities, but also radiates the energy efficiency improvement of neighboring cities.

This paper can be regarded as an attempt to explore how im-porting intermediate goods may affect energy efficiency. It deepens the research pertaining to China’s import trade and urban energy governance, and provides empirical evidence on the impact of intermediate goods imports on energy efficiency, which have significant implications. The findings of this paper highlight the importance of imported intermediate goods in improving energy efficiency and provide new perspectives on the development of regional energy governance systems. The findings of this paper are applicable to some developing countries. Due to a large technological gap between the developing and developed countries, high-quality, multi-species imported intermediate goods can have a technological spillover effect and complementary effect on the host country, thus improving energy efficiency. However, this improvement can be affected by various factors, such as absorptive capacity. Therefore, the effect of energy efficiency of imported intermediate goods depends on the real situation of the host country.

## Figures and Tables

**Table 1:** Meaning and measurement of energy efficiency indicators.

Indicators	Categories	Specific Composition	Unit
Input Desired output Non-desired output	Labor Capital Energy	employees at the end of the year Capital stock calculated using the perpetual inventory method Consumption of electricity and liquefied petroleum gas	10,000 Million yuan Tons of standard coal
	Economic output Pollution emissions	Real GDP calculated using 2000 as the base period CO_2_, SO_2_	Million yuan Tons

**Table 2:** Impact of imports of intermediate goods on energy efficiency.

	(1)	(2)	(3)	(4)	(5)
	EE	EE	EE	EE	EE
lninter	0.377 ***	0.145 ***	0.140 ***	0.175 ***	
	(0.0186)	(0.0183)	(0.0156)	(0.0177)	
L. lninter					0.155 ***
					(0.0175)
lnpgdp		0.154 ***	0.0848 ***	0.134 ***	0.239 ***
		(0.00419)	(0.00775)	(0.00990)	(0.0130)
lnfdi		0.00523 ***	−0.00166	−0.00101	−0.00119
		(0.00200)	(0.00168)	(0.00175)	(0.00175)
urban		−0.0125	0.000271	−0.0712 ***	−0.0693 ***
		(0.00961)	(0.00911)	(0.0244)	(0.0245)
fina		−0.0619 ***	−0.0334 ***	−0.0188 ***	−0.0107 *
		(0.00473)	(0.00443)	(0.00522)	(0.00552)
RF		−0.00338 *	−0.000512 *	−0.000576 **	−0.00117 *
		(0.00225)	(0.00184)	(0.00185)	(0.00178)
Constant	0.336 ***	−1.026 ***	−0.524 ***	−0.949 ***	−1.947 ***
	(0.00884)	(0.0376)	(0.0654)	(0.0867)	(0.118)
Year FE	No	No	Yes	Yes	Yes
City FE	No	No	No	Yes	Yes
Observations	4624	4571	4571	4571	4320
R^2^	0.104	0.422	0.609	0.584	0.574

Note: Robust standard errors in parentheses; *** *p* < 0.01, ** *p* < 0.05, * *p* < 0.1; clustered at city levels.

**Table 3:** Quantile regression.

	(1)	(2)	(3)	(4)	(5)
	0.10	0.25	0.50	0.75	0.90
lninter	0.0861 *** (0.0889)	0.0919 *** (0.1181)	0.152 ** (0.0219)	0.216 *** (0.0223)	0.247 *** (0.0474)
Constant	0.099 *** (0.261)	0.075 *** (0.256)	0.110 *** (0.029)	0.074 *** (0.044)	0.156 * (0.1186)
Control variables	Yes	Yes	Yes	Yes	Yes
Year FE	Yes	Yes	Yes	Yes	Yes
City FE	Yes	Yes	Yes	Yes	Yes
N	2200	2200	2200	2200	2200
R^2^	0.103	0.227	0.351	0.304	0.210

Note: Robust standard errors in parentheses; *** *p* < 0.01, ** *p* < 0.05, * *p* < 0.1; clustered at city levels.

**Table 4:** Test of instrumental variables method.

	Intermediate Goods Imports Tariff		Distance to Port	
	(1) Phase I	(2) Phase II	(3) Phase I	(4) Phase II
IV-lninter	−0.00575 *** (0.000470)		−0.0572 *** (0.00115)	
lninter		0.8352 *** (0.1399)		1.0517 *** (0.0760)
Controls	Yes	Yes	Yes	Yes
City FE	Yes	Yes	Yes	Yes
Year FE	Yes	Yes	Yes	Yes
Kleibergen-Paap rk LM statistic	155.880 [0.000]		23.467 [0.000]	
Kleibergen-Paap Wald rk F statistic	149.832 {16.38}		24.758 {16.38}	
N	4624	4624	4624	4624
R^2^	0.503	0.503	0.526	0.526

Note: Robust standard errors in parentheses; *** *p* < 0.01, lustered at city levels middle brackets is p-value, the critical value of Stock-Yogo at 10% in the brace; original hypothesis of Kleibergen-Paap rk LM statistic is that instrumental variables are underidentified; original hypothesis of Kleibergen-Paap Wald rk F statistic is the presence of weak instrumental variables.

**Table 5:** Robustness test.

	PTEE	Excluding Macro-Systematic Differences	Data Tailing	Excluding Impact of Energy Policies
lninter	2.74 ***	0.169 ***	0.157 ***	0.148 ***
	(4.056)	(0.0159)	(0.0161)	(0.0296)
Constant	0.572***		1.228***	1.662***
	(0.0437)		(0.134)	(0.129)
Control variables	Yes	Yes	Yes	Yes
Province × Year FE	No	Yes	No	No
City FE	Yes	Yes	Yes	Yes
Year FE	Yes	Yes	Yes	Yes
N	4253	4539	4532	3825
R^2^	0.163	0.523	0.638	0.608

Note: Robust standard errors in parentheses; *** *p* < 0.01; clustered at city levels.

**Table 6:** Mechanism test.

	(1)	(2)	(3)
	EE	EE	EE
lninter	0.139 ***	−0.0621	0.0888
	(0.0225)	(0.117)	(0.137)
Lninter × lnspillover	0.124 ***		0.103 **
	(0.0397)		(0.0482)
Lninter × types		0.0252 **	0.00518
		(0.0125)	(0.0156)
Constant	−1.638 ***	−1.160 ***	−1.174 ***
	(0.116)	(0.121)	(0.121)
Control variables	Yes	Yes	Yes
City FE	Yes	Yes	Yes
Year FE	Yes	Yes	Yes
N	4624	4407	4407
R^2^	0.588	0.601	0.602

Note: Robust standard errors in parentheses; *** *p* < 0.01, ** *p* < 0.05; clustered at city levels.

**Table 7:** The impact of geographic location.

	(1)	(2)	(3)	(4)	(5)
	East	Central	West	South	North
lninter	0.125 ***	0.122 *	0.0504	0.144 **	0.209 ***
	(0.0538)	(0.0714)	(0.113)	(0.0636)	(0.0556)
	(0.0164)	(0.0144)	(0.00307)	(0.00379)	(0.00146)
Constant	−0.226	−0.776 **	−2.517 ***	−0.514	−1.710 ***
	(0.640)	(0.379)	(0.596)	(0.526)	(0.413)
Controls	Yes	Yes	Yes	Yes	Yes
Year FE	Yes	Yes	Yes	Yes	Yes
City FE	Yes	Yes	Yes	Yes	Yes
N	1428	1292	1326	2329	2091
R^2^	0.684	0.666	0.562	0.604	0.663

Note: Robust standard errors in parentheses; *** *p* < 0.01, ** *p* < 0.05, * *p* < 0.1; clustered at city levels.

**Table 8:** The impact of geographical features and resource endowment.

	Relief Amplitude			Resource Endowment	
	(1)	(2)	(3)	(4)	(5)
	Low	Middle	High	Resource Cities	Other Cities
lninter	0.182 ***	0.148 **	0.0291	0.137 ***	0.348 ***
	(0.0578)	(0.0666)	(0.0888)	(0.0511)	(0.0846)
Constant	−0.639	−0.621	−2.709 ***	−1.664 ***	−1.259 ***
	(0.527)	(0.534)	(0.751)	(0.638)	(0.325)
Controls	Yes	Yes	Yes	Yes	Yes
Year FE	Yes	Yes	Yes	Yes	Yes
City FE	Yes	Yes	Yes	Yes	Yes
N	1530	1547	1547	1836	2788
R^2^	0.717	0.593	0.562	0.656	0.594

Note: Robust standard errors in parentheses; *** *p* < 0.01, ** *p* < 0.05; clustered at city levels.

**Table 9:** The impact of population scale.

	(1)	(2)	(3)	(4)	(5)
	Small City	Medium City	Large City	Megacity	Super Megacity
lninter	0.803	0.196	0.294 ***	0.0730 **	0.0979 *
	(2.834)	(0.214)	(0.0315)	(0.0294)	(0.0530)
Constant	−0.539	−1.273	−1.574 ***	−0.164	−2.873 **
	(2.600)	(0.817)	(0.0824)	(0.250)	(1.231)
Controls	Yes	Yes	Yes	Yes	Yes
Year FE	Yes	Yes	Yes	Yes	Yes
City FE	Yes	Yes	Yes	Yes	Yes
N	49	144	2895	1371	165
R^2^	0.481	0.387	0.588	0.636	0.602

According to “The Notice on Adjusting the Criteria for the Classification of City Scale” issued by China State Council in 2014, small city: permanent population less than 500,000, medium city: permanent population of more than 500,000 and less than 1 million; large city: permanent population of more than 1 million and less than 5 million s, megacity: permanent population of more than 5 million and less than 10 million; super megacity: permanent population of 10 million. Robust standard errors in parentheses; *** *p* < 0.01, ** *p* < 0.05, * *p* < 0.1; clustered at city levels.

**Table 10:** The impact of absorptive capacity.

	Human Capital			R&D		
	(1)	(2)	(3)	(4)	(5)	(6)
	Low	Middle	High	Low	Middle	High
lninter	0.201 *	0.164 **	0.132 ***	0.0233	0.0403	0.227 ***
	(0.106)	(0.0683)	(0.0216)	(0.0271)	(0.0409)	(0.0533)
Constant	−2.370 ***	−0.880 ***	−0.600 ***	−2.111 ***	−0.188	−1.449 ***
	(0.201)	(0.236)	(0.201)	(0.256)	(0.205)	(0.288)
Controls	Yes	Yes	Yes	Yes	Yes	Yes
Year FE	Yes	Yes	Yes	Yes	Yes	Yes
City FE	Yes	Yes	Yes	Yes	Yes	Yes
N	1530	1547	1547	1351	1362	1358
R^2^	0.446	0.538	0.666	0.453	0.556	0.705

Note: Robust standard errors in parentheses; *** *p* < 0.01, ** *p* < 0.05, * *p* < 0.1; clustered at city levels.

**Table 11:** Spatial correlation analysis: global Moran index by year.

**Year**	$W_{1}$	$W_{2}$	$W_{3}$
2000	0.003	0.020 *	0.001
2001	0.003	0.008	0.001
2002	0.010	0.020 *	0.001
2003	0.005 *	0.001 *	0.003
2004	0.167 ***	0.185 ***	0.082 ***
2005	0.113 ***	0.138 ***	0.065 ***
2006	0.140 ***	0.171 ***	0.057 ***
2007	0.104 ***	0.142 ***	0.032 ***
2008	0.114 ***	0.157 **	0.025 ***
2009	0.091 ***	0.103 ***	0.022 ***
2010	0.084 ***	0.091 ***	0.017 ***
2011	0.083 ***	0.069 **	0.030 ***
2012	0.106 ***	0.086 ***	0.051 ***
2013	0.075 ***	0.082 **	0.014 ***
2014	0.090 ***	0.086 **	0.016 ***
2015	0.072 ***	0.090 ***	0.008 *
2016	0.118 ***	0.116 ***	0.023 ***

Note: *** *p* < 0.01, ** *p* < 0.05, * *p* < 0.1.

**Table 12:** Spatial measures of the impact of intermediate goods imports on energy efficiency.

	SDM			SAR		
	(1)	(2)	(3)	(4)	(5)	(6)
	*W*_1_	*W*_2_	*W*_3_	*W*_1_	*W*_2_	*W*_3_
lninter	0.235 ***	0.180 ***	0.178 ***	0.136 ***	0.156 ***	0.129 ***
	(0.0195)	(0.0175)	(0.0184)	(0.0179)	(0.0184)	(0.0176)
W × lninter	0.118 ***	0.146	0.120 ***			
	(0.0345)	(0.125)	(0.0463)			
Spatial rho	0.651 ***	0.899 ***	0.812 ***	0.653 ***	0.468 ***	0.851 ***
	(0.0126)	(0.0149)	(0.0151)	(0.0171)	(0.0149)	(0.0187)
Control variables	Yes	Yes	Yes	Yes	Yes	Yes
W × Control variables	Yes	Yes	Yes	Yes	Yes	Yes
City FE	Yes	Yes	Yes	Yes	Yes	Yes
Year FE	Yes	Yes	Yes	Yes	Yes	Yes
Direct effect	0.272 ***	0.194 ***	0.198 ***	0.141 ***	0.162 ***	0.132 ***
	(0.0201)	(0.0177)	(0.0188)	(0.0184)	(0.0191)	(0.0180)
Indirect effect	0.734 ***	3.179**	1.376 ***	0.252 ***	0.130 ***	0.744 ***
	(0.0690)	(1.450)	(0.168)	(0.0321)	(0.149)	(0.121)
Total effect	1.006 ***	3.373 **	1.574 ***	0.393 ***	0.292 **	0.877 ***
	(0.0765)	(1.453)	(0.174)	(0.0488)	(0.332)	(0.132)
N	4624	4624	4624	4624	4624	4624
R^2^	0.2138	0.2551	0.4839	0.4197	0.4235	0.4057
	Model Comparison					
Wald spatial lag	6.71 ***	11.75 ***	15.21 ***			
	(0.0096)	(0.0006)	(0.0001)			
LR spatial lag	6.75 ***	11.80 ***	13.18***			
	(0.0094)	(0.0003)	(0.0003)			
Wald spatial error	38.94 **	76.11 ***	57.85 ***			
	(0.0000)	(0.0000)	(0.0001)			
LR spatial error	39.49 ***	76.41 **	60.37 **			
	(0.0000)	(0.0000)	(0.0000)			

Note: Robust standard errors in parentheses; *** *p* < 0.01, ** *p* < 0.05; clustered at city levels; original hypothesis for the Wald and LR spatial lag term tests: the SDM model can be reduced to a SAR model; original hypothesis for the Wald and LR spatial error term tests: the SDM model can be reduced to a SEM model. *p*-values in parentheses below the Wald and LR test statistics.

## Data Availability

The data presented in this study are available on request from the authors.
